# 2321. Prevalence and determinants of depression among healthcare workers during the COVID-19 pandemic in Khujand, Tajikistan, 2022

**DOI:** 10.1093/ofid/ofad500.1943

**Published:** 2023-11-27

**Authors:** Jamila Silemonshoeva, Roberta Horth, Zulfiya Haybullo Tilloeva, Navruz Jafarov, S A N A M ZIKRIYAROVA, Dilyara Nabirova

**Affiliations:** Central Asia Field Epidemiology Training Program, Khujand, Sughd, Tajikistan; US Centers for Disease Control and Prevention, Dulles, Virginia; City Desinfection station, Dushanbe, Republic of Tajikistan, Dushanbe, Dushanbe, Tajikistan; Ministry of Health, Dushanbe, Dushanbe, Tajikistan; Kazakh National Medical University named after S.D. Asfendiyarov;, Almaty, Almaty, Kazakhstan; CDC Central Asia office, Almaty, Almaty, Kazakhstan

## Abstract

**Background:**

Healthcare workers experienced substantial work overload and stress during the COVID-19 response. Increase in prevalence of mental health conditions among healthcare workers have been reported globally. Low-resource settings, such as Tajikistan, experienced additional challenges related to the COVID-19 pandemic and have scare resources to mitigate mental health consequences. To inform program planning, we aimed to determine prevalence of depression, associated factors, and care gap among healthcare workers.

**Methods:**

We conducted a cross-sectional study of all healthcare providers (physicians, nurses, and nurse assistants) working from July to August 2022 in three COVID-19 referral hospitals and three polyclinics in Khujand city, Tajikistan. We systematically sampled every fifth provider. Depression was assessed using the Hospital Anxiety and Depression Scale. We used multivariable logistic regression model adjusting for random effect of hospital to identify factors associated with depression.

**Results:**

Among 400 healthcare workers, 277 (69%) had depression (Table 1). Half (44%) no longer enjoyed the things they used to (Figure 1). Prevalence of depression was significantly higher (p< 0.01) among females than males (71% vs 59%), nurses than physicians (77% vs 56%). Among providers with depression, 20% had received professional services, 32% had taken a vacation, 32% had severe COVID-19, and 53% experienced frequent respiratory issues, and 56% felt people avoided them because of work. In multivariable analysis depression was associated with being a nurse (adjusted odds ratio [aOR]=3.5; 95% confidence ratio [CI]: 1.7-7.2), having had severe COVID-19 disease (aOR=4.0; 95% CI: 1.4-11.8) and having felt that people avoided them because of work (aOR=1.6; 95% CI: 1.0-2.6) (Table 2). Providers who used respirators >3 hours had lower odds of depression (aOR=0.4; 95% CI: 0.2-0.9).

General characteristics of healthcare providers by depression, Tajikistan, 2022

**Figure:**
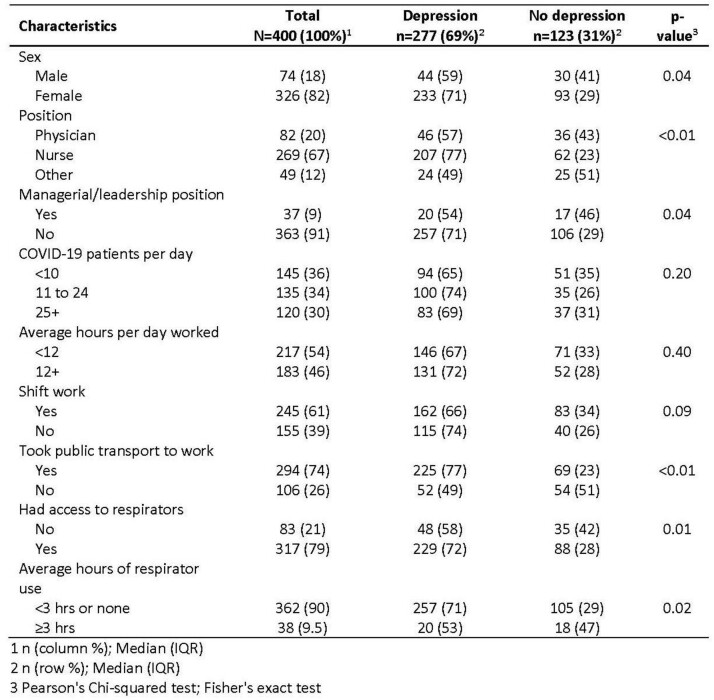

External pressures, physical conditions and supportive services among and depression among healthcare workers, Tajikistan, 2021

**Figure:**
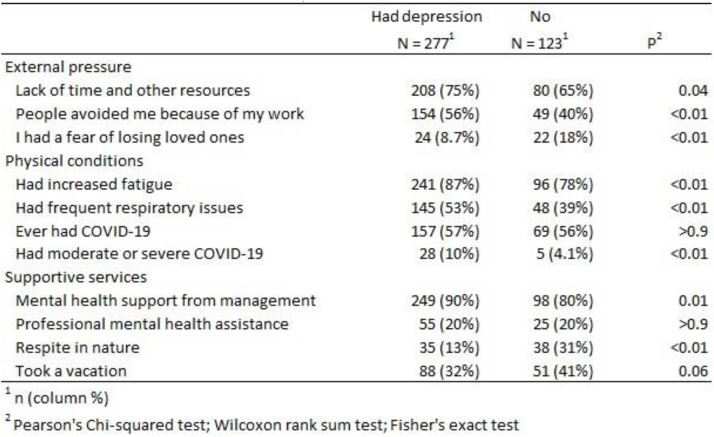

Factors associated with depression among healthcare workers, Tajikistan, 2021

**Figure:**
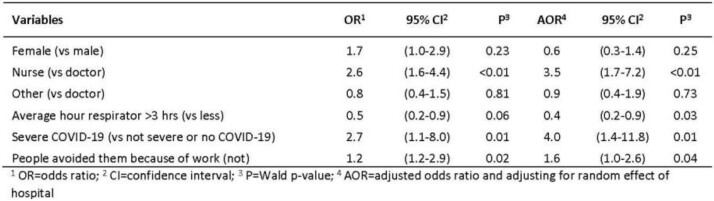

**Conclusion:**

We found high prevalence of depression among healthcare workers in Khujand city, Tajikistan. Timely screening and increase in provision of supportive services for providers is needed. These services should be offered to all providers, and especially targeted towards those with prior severe COVID-19 disease and nurses.

**Disclosures:**

**All Authors**: No reported disclosures

